# Evidence of the content validity, acceptability, and feasibility of a new Patient-Reported Impact of Dermatological Diseases measure

**DOI:** 10.3389/fmed.2023.1020523

**Published:** 2023-05-05

**Authors:** Rachael Pattinson, Nirohshah Trialonis-Suthakharan, Rachael M. Hewitt, Maria José Valencia López, Nasim Tahmasebi Gandomkari, Jennifer Austin, Allison FitzGerald, Nick Courtier, Matthias Augustin, Chris Bundy

**Affiliations:** ^1^School of Healthcare Sciences, Cardiff University, Cardiff, Wales, United Kingdom; ^2^Institute for Health Services Research in Dermatology and Nursing (IVDP), University Medical Center Hamburg-Eppendorf (UKE), Hamburg, Germany; ^3^International Alliance of Dermatology Patient Organizations, Ottawa, ON, Canada

**Keywords:** patient-reported outcome measure, dermatology, pilot test, cognitive interview, content validity (MeSH), patient-centered, quality of life, burden of disease

## Abstract

**Background:**

The Global Research on the Impact of Dermatological Diseases (GRIDD) team is developing the new Patient-Reported Impact of Dermatological Diseases (PRIDD) measure of the impact of dermatological conditions on the patient’s life, in partnership with patients. To develop PRIDD, we conducted a systematic review, followed by a qualitative interview study with 68 patients worldwide and subsequently a global Delphi survey of 1,154 patients to ensure PRIDD items were meaningful and important to patients.

**Objective:**

To pilot test PRIDD with patients with dermatological conditions, focusing on its content validity (comprehensiveness, comprehensibility, and relevance), acceptability, and feasibility.

**Methods:**

We conducted a theory-led qualitative study using the Three-Step Test-Interview method of cognitive interviewing. Three rounds of semi-structured interviews were conducted online. Adults (≥ 18 years) living with a dermatological condition and who spoke English sufficiently to take part in the interview were recruited through the International Alliance of Dermatology Patient Organizations’ (GlobalSkin) global membership network. The topic guide met the gold-standard COSMIN (Consensus-based Standards for the Selection of Health Measurement Instruments) standards for cognitive interviewing. Analysis followed the thematic analytical model of cognitive interviewing.

**Results:**

Twelve people (58% male) representing six dermatological conditions from four countries participated. Overall, patients found PRIDD to be comprehensible, comprehensive, relevant, acceptable, and feasible. Participants were able to discern the conceptual framework domains from the items. Feedback resulted in: the recall period being extended from 1 week to 1 month; removal of the ‘not relevant’ response option; and changes to the instructions and item ordering and wording to improve clarity and increase respondents’ confidence in their ability to respond. These evidence-based adjustments resulted in a 26-item version of PRIDD.

**Conclusion:**

This study met the gold-standard COSMIN criteria for the pilot testing of health measurement instruments. The data triangulated our previous findings, in particular the conceptual framework of impact. Our findings illuminate how patients understand and respond to PRIDD and other patient-reported measurement instruments. The results of comprehensibility, comprehensiveness, relevance, acceptability, and feasibility of PRIDD provide evidence of content validity from the target population. The next step in the development and validation of PRIDD is psychometric testing.

## 1. Introduction

Dermatological conditions carry a substantial physical, psychological, and social burden for patients (1, 2). The stigma of living with a visible condition (3), symptoms including pain and itch (4, 5), and financial cost (6) partially explain this burden (7). Many dermatological conditions have associated comorbidities (8), further increasing the disease burden (9).

The Global Burden of Disease (GBD) project (10, 11) is the most comprehensive worldwide epidemiological study to date, providing burden and mortality estimates for health problems at global, national, and regional levels. These estimates are exceptionally influential as they provide the evidence-base for identifying patient need, determining resource allocation and research priorities globally. We, along with others in the dermatology community [e.g., (12)], maintain that the GBD studies systematically underestimate the burden of dermatological conditions as they are evaluated according to symptoms that affect only the skin (itch, disfigurement) and do not include the broader psychological and social aspects such as depression, anxiety, stigma, and social isolation in their measure of impact (13–16).

The Global Research on the Impact of Dermatological Diseases (GRIDD) project, the first patient-initiated and led impact research project in dermatology, is collecting global data on the impact of living with dermatological conditions. These data will support local, national, and international advocacy work to prioritize dermatological conditions more accurately in the global health debate.

To address GRIDD’s aim, a comprehensive measure of the impact of dermatological conditions on the patient’s life is required. Our systematic review (17) evaluated the quality of existing dermatology-specific (can be used across conditions) patient-reported outcome measures (PROMs) against the gold-standard Consensus-based Standards for the Selection of Health Measurement Instruments (COSMIN) criteria (18). PROMS, like all measurement instruments (e.g., thermometers, sphygmomanometers), must meet predefined criteria for measurement properties—validity, reliability, and responsiveness—to have confidence that the data they produce are accurate (19–21). None of the 36 existing dermatology PROMs identified in our review, including widely used measures such as Dermatology Life Quality Index (DLQI) (22) and Skindex (23–25), met the standards to be recommended for use according to their known measurement properties and could not capture the full impact of the dermatological condition on the patient’s life according to our conceptual framework of impact (26). The single most common reason for poor quality assessment was insufficient patient involvement during PROM development. This included, for example, inadequate sample sizes and inappropriate data collection methods (27, 28). Other systematic reviews of existing quality of life PROMs in the context of psoriasis (29), eczema (30), and acne (31) have found a similar lack of adequate dermatology-specific PROMs.

We are developing the Patient-Reported Impact of Dermatological Diseases (PRIDD) measure in close collaboration with patients and according to best practices in PROM development (Supplementary material 1) (18, 19, 32–34). PRIDD is designed for use with adults (≥18 years) with a dermatological condition worldwide and for use in research and clinical practice. Congruent with best practice in cross-cultural translation of PROMs, PRIDD is initially being developed and validated in English before being translated into other languages.

Content validity, “the degree to which the content of an instrument is an adequate reflection of the construct to be measured” (35), is considered the most important measurement property (28, 36). We began the content validity phase of PRIDD development by conducting a qualitative interview study with 65 patients from 29 countries representing 29 dermatological conditions (26) and identified 263 areas of impact that cut across conditions and global regions. This work formed, to our knowledge, the first conceptual framework of the impact of dermatological conditions on patients’ lives. In the second phase of PRIDD development, 1,154 patients from 65 countries representing 90 dermatological conditions participated in a global Delphi survey to prioritize the 263 items for inclusion in PRIDD (37). While existing dermatology PROMs have included a range of domains relevant to the construct of impact (17), no single PROM has unified all relevant domains outlined in the conceptual framework of impact. This demonstrates that, as the first measure to capture all aspects of the conceptual framework as a unified construct, PRIDD advances knowledge in and makes a unique contribution to dermatology.

Following best practice, the next phase in PRIDD development is to pilot test the measure with dermatology patients and make any necessary adjustments (38, 39). The purpose of the pilot is to rigorously test three aspects of content validity: comprehensiveness (all key aspects of impact are present), comprehensibility (items are understood by respondents as intended) and relevance (all items are relevant to the impact of dermatological conditions from the patients’ perspective) (39). The acceptability (whether patients are willing to complete the instrument) and feasibility (whether patients are able to complete the instrument) can also be tested.

Cognitive interviewing is a pilot testing method that use a semi-structured topic guide to direct the interview according to Tourangeau’s four-stage model of question response (40, 41) to obtain information about how participants interpret questions and choose their answers. The COSMIN group recommend the Three-Step Test-Interview (TSTI) (42, 43) method of cognitive interviewing as this combines the “think-aloud” (44) and “probing” techniques (36, 38), thereby offsetting the weaknesses of each and providing a deeper understanding of how questions are interpreted and answered (38).

The aim of the current study was to pilot-test PRIDD by qualitatively exploring whether the measure (items, structure, response options and recall period) is comprehensive, comprehensible, relevant, feasible, and acceptable to people with dermatological conditions through cognitive interviews.

## 2. Materials and methods

### 2.1. Design

We conducted a theory-led, qualitative study using the TSTI method of cognitive interviewing to pilot test PRIDD. This study was tested against the latest COSMIN guidance on the pilot testing of PROMs (27, 33, 45) and is reported according to the Cognitive Interviewing Reporting Framework (46). Ethical approval was obtained from Cardiff University School of Healthcare Sciences Ethics Committee (SREC:637).

### 2.2. Participants

Participants met the inclusion criteria if they were an adult (≥18 years) with a dermatological condition from anywhere in the world and spoke English sufficiently to take part in the interview and complete PRIDD independently (without a translator). Those who required translation to complete PRIDD were excluded as construct equivalence, the assumption that items in the translation measure the same construct in the same way as in the original language (47–49), could not be determined and, therefore, confidence in the evidence of content validity would be lacking. Children and proxies, such as family members or carers, were also excluded as they are not PRIDD’s target population.

Participants were drawn from PRIDD’s target population via the International Alliance of Dermatology Patient Organizations’ (GlobalSkin) global membership network using purposive sampling to achieve maximum variation according to dermatological condition and demographic factors: age, gender, and country of residence. Participants were directed to a secure online platform which included the participant information sheet (PIS), electronic consent form, and interview booking information. Twelve patient organizations were invited to recruit to the interviews; 8 (66.7%) agreed to participate. Reasons for non-participation included lack of staff capacity, scheduling conflicts, and non-response. Recruitment ceased at the point of data saturation; when there was sufficient evidence that most problems had been detected and/or resolved (46, 50).

### 2.3. Materials

We tested the first draft of PRIDD (Supplementary material 2), a 27-item, English language measure of the impact of a dermatological condition on the patient’s life over the last week. The conceptual framework of impact (26) depicts a reflective measurement model (Supplementary material 3). The first draft of PRIDD has five subscales: physical impact, psychological impact, social impact, daily life and responsibilities impact, and financial impact. All items are rated on a 5-point scale with scores of 0 (“never”), 1 (“rarely”), 2 (“sometimes”), 3 (“often”), and 4 (“always”), each with an additional “not relevant” option.

A topic guide was developed detailing the interview procedures, instructions, and questions (Supplementary material 4). Two versions of PRIDD were used during the interviews: one with the original item order and one with items in reverse order. This enabled us to test item order effects (i.e., whether the order in which the items are presented affects people’s responses) and establish whether items were understood independently of each other. An online platform was created to enroll potential participants in the study using the PIS and consent form and included a demographic questionnaire.

### 2.4. Procedure

Twelve interviews were conducted via Zoom video conferencing software across three rounds from 2 August to 1 September 2021 with one of four researchers (RP, RH, MVL, and NTG), at a mutually convenient time. All interviewers were trained in cognitive interviewing by the study co-investigator (CB) and had backgrounds in healthcare practice and/or research.

Interviews were approximately 1 h long and followed the three steps of the TSTI method (see Supplementary material 5). They were audio-recorded using a high-quality audio recorder (OLYMPUS WS-833) and transcribed verbatim. Transcripts were checked and anonymized by RP by being allocated participant identifiers (PIDs).

### 2.5. Analysis

Data collection and analysis were interrelated and concurrent, with analysis beginning after the completion of the first interview. Accordingly, generated themes and edits made to PRIDD were incorporated into subsequent interviews.

Quantitative data were uploaded to SPSS version 26 and sample characteristics were summarized for clinical and demographic variables. Qualitative data were exported to NVivo 12 qualitative data software package. RP independently analyzed the data. NTS reviewed the coding and results reporting for accuracy. Analysis followed the thematic analytical model of cognitive interviewing (51) (see Supplementary material 6). This produced a summary of each item’s performance that established the comprehensiveness, comprehensibility, and relevance of the items, providing evidence of content validity and informing evidence-based improvements.

## 3. Results

Eighteen people completed the online consent form and demographics questionnaire. Of these, three people were excluded because they were not sufficiently proficient in English to complete PRIDD independently and three did not respond to invitations to schedule an interview. In total, 12 people (response rate = 67%) across six dermatological conditions (Table 1) and four countries participated in an interview.

**Table 1:** Participant characteristics.

	*n* (%)
Total	12
Age	*M* = 53.42 (SD = 15.87, range = 29–75)
Gender	
Male	7 (58.3)
Female	5 (41.7)
Dermatological condition	
Common^a^	7 (58.3)
Rare^b^	5 (41.7)
Duration (years)	*M* = 31.39 (SD = 20.59, range = 3–60)
Country	
UK	7
Ireland	3
Canada	1
USA	1

a Psoriasis (*n* = 5); Alopecia (*n* = 2).

b Discoid Lupus, Hidradenitis Suppurativa, Extensive Linear Porokeratosis (*n* = 1); Pityriasis Rubra Pilaris (*n* = 2).

Supplementary material 7 outlines the changes made to PRIDD between the three rounds of cognitive interviews. Evidence-based adjustments resulted in a 26-item version of PRIDD (Supplementary material 8).

### 3.1. General feedback

Participants praised the comprehensiveness, comprehensibility, and relevance of PRIDD to their lived experiences:

I’ve completed a lot of dermatological questionnaires, but I don’t think I’ve ever seen them all integrated like this in such a questionnaire … I’m very, very happy with this. It has stirred my heart … There are things here that I wanted to discuss with my dermatologist. PID5, Patient with hidradenitis suppurativa, Ireland

They were short questions, and they were quite easily answered. PID15, Patient with alopecia, UK

PRIDD also appeared to be acceptable and feasible for patients. The average time taken to complete the questionnaire was 4.11 min (SD = 1.35, range = 2.62–7).

The questions are so concise. You can quickly fill that in, in the waiting room. PID12, Patient with extensive linear porokeratosis, Ireland

While no participants found any of the items offensive or objectionable, they felt that others might be “uncomfortable” (PID9, Patient with psoriasis, UK) with item 26 (“it has been difficult to be intimate with a partner”) as it referred to intimacy, but stressed it was important to include. Instead, participants felt that completing PRIDD initiated a process of reflection on their experiences with their condition:

I wasn’t offended by any of them [items] … It actually made me aware of how much this is actually controlling my life again. PID11, Patient with discoid lupus, Ireland

A minority of participants, most with alopecia, questioned the focus on the negative impacts of dermatological conditions and felt that positive impacts should be included too.

I think it's sometimes nice to balance the negatives out with positives … in the past week, I've felt a lot of empowerment, I've felt a lot of like confidence, I've felt a lot of people praising me for something I've tried to hide away for so long, so it's not just negatives that you could capture as well, having a separate question saying I've felt confident, or I've felt empowered or something. PID15, Patient with alopecia, UK

Some participants wanted to further elaborate on items with qualitative data.

You could even go deeper than that … you could even have … a box to maybe put is there anything you'd like to add … that [you] feel is relevant … because everybody isn't the same PID16, Patient with Pityriasis Rubra Pilaris (PRP), UK

### 3.2. Feedback on instructions

Overall, the instructions appeared easy to comprehend as participants were able to summarize them accurately and succinctly.

It was asking me to answer the below questions based on my condition, how it's affected me in the last week, and answer them with what's relevant to me and my experience. … I felt the instructions were really clear … it's a fairly straightforward questionnaire PID15, Patient with alopecia, UK

However, some “did not read that part [instructions]” (PID11, Patient with discoid lupus, Ireland), which affected the validity of their answers, particularly in relation to the recall period. On this basis, several sections of the instructions were emboldened to draw respondents’ attention to the instructions and their most important aspects.

Some suggestions to improve clarity were provided. First, participants felt that the example “because you do not work” created confusion as it led participants to believe that the items should be answered in relation to both their dermatological condition and employment. As a result, this example was removed from the instructions.

Going through the questions in my head, I don’t know how work would have anything to do with the questions that were asked … I don’t even really think you need it all. PID14, Patient with psoriasis, Canada

Second, some participants suggested alternatives to the term “dermatological conditions,” feeling it was wordy. A minority of participants with conditions primarily affecting the skin suggested using ‘a simple word like skin’ (PID9, Patient with psoriasis, UK) instead. Others with conditions that did not primarily affect the skin such as alopecia felt that dermatology implied a focus on the skin and would prefer another word, but could not provide a suitable alternative:

Is there another word to say skin, which includes hair, nails, whatever, you know? I don't think there is, but that's the only thing that I would maybe look into, but I don't think there is a synonym. PID18, Patient with alopecia, UK

Because most participants found it acceptable and it is more inclusive than “skin,” the term ‘dermatological conditions’ was retained.

You need it to be applicable to several different conditions, not just one and … there is the difficulty. So with that in mind, your questions are brilliant. PID18, Patient with alopecia, UK

I am sure phrases like dermatological, I mean will be familiar to anyone with any sort of conditions PID13, Patient with psoriasis, UK

### 3.3. Feedback on the recall period

Participants were almost unanimous in their criticism of the one-week recall period. Many felt that a longer recall period was required to accurately reflect the impact that their dermatological condition has had on their lives, largely due to the relapse and remitting nature of many of these conditions:

Seven days isn’t long enough for someone with … [a] condition that they’ve no control over, and people can see it. Because that’s another thing like, lupus can flare, and it’ll go back down, and I can have three good weeks and then one really crap week where it’s just blown up on my face. So, I still think that the past week is too short a term to ask someone how it is. PID11, Patient with discoid lupus, Ireland

Participants also explained that many people do not engage in some of the experiences captured by the items (e.g., intimacy, life decisions and social activities) on a weekly basis and so expanding the recall period would likely increase item relevance to a higher proportion of patients and consequently more accurately capture disease burden:

If the timeframe had been three months, six months, a year, or your lifetime … the feedback would be very different. So, you talk about relationships, intimate with [a] partner, all of these kinds of things, you know, social interactions, if somebody hasn’t had a social interaction in the last week they’re going to say never, whereas if the timeframe is much larger, you’re going to get a more realistic feedback. PID12, Patient with extensive linear porokeratosis, Ireland

A 2-week and 1-month recall period were tested. These were generally more acceptable to participants than the 1-week recall. A 1-month recall period was adopted, having been suggested as an alternative to the 1-week recall period by multiple participants.

### 3.4. Feedback on the items

A summary of the evidence of comprehensibility, relevance, and problems detected for each item is presented in Supplementary material 9. Nine of the 27 items remained unchanged because they were easily understood, relevant to participants and distinct from other items.

Sixteen items were modified to align them more closely with the intended concept of interest outlined in the item definition list or to reduce conceptual overlap with other items.

One item, “I have felt dismissed or abandoned by others,” was deleted because it was not easily understood by participants and was felt to be highly similar to “I have been excluded, bullied or discriminated against.”

Overall, participants found the item ordering acceptable. The five domains of the conceptual framework were evident in the items, as participants correctly recognized categories of items, providing evidence in support of the suitability of the item ordering as well as the conceptual framework.

They seemed to be grouped together quite well and I think the order of them was fine. PID15, Patient with alopecia, UK

The order of seven items was changed to enhance understanding. For example, the item “my everyday choices have been affected (for example, choice of clothes, hairstyle or products)” was listed before the item “my life goals and choices have been affected (for example, career choice or having children)” to highlight that the latter does not include everyday choices, which some participants subsumed under life goals and choices.

Subgroup differences were found on four items. People with alopecia felt that the item “my treatment has caused practical problems (for example, by taking up time or being messy)” was not relevant to them as they had no treatments. They also differed from people with other conditions on three items as alopecia appeared to have a positive impact in terms of timesaving, reduced financial costs and feelings of attractiveness.

It positively impacted it [daily routine], because I don't have to mess about with my hair as much in the morning… it's a bit of a blessing. PID15, Patient with alopecia, UK

### 3.5. Feedback on the response options

Participants found the response options (“never,” “rarely,” “sometimes,” “often,” and “always”) to be appropriate, cover the full range of experience, and comprehensible.

I found it quite easy, I think it gives a good range of options. Obviously always and never are complete extreme [s] and then a couple of the intermediates of different intensity, is fine. I think it is a really good way of asking questionnaires and usually makes it quite easy to answer. PID13, Patient with psoriasis, UK

Some participants had difficulty interpreting the “not relevant” response (NRR) option. NRRs caused confusion, especially for items where the condition had been an obstruction to engaging in the relevant life area. This was most clearly discussed in relation to the item on intimacy, for example:

‘It's been difficult to be intimate with a partner’ … this is the question that I always struggle with. [I chose] not relevant because I've not got a partner, but that can also be always, because I haven't got a partner, it can be both … the reason why I've not got a partner is because it's been difficult … I think it's an important question … I think [psoriasis is] … probably the reason why I'm single. In my formative years between when I started getting psoriasis and in hospital, was years when all my mates were getting wives and babies and all that. All of a sudden it had just passed me by, it had gone, you know. And it's like all of a sudden, I'm 40 odd and I'm like all my mates are married and having kids and I seem to have missed that bit. PID1, Patient with psoriasis, UK

Others could not distinguish the NRR option from the ‘never’ response option.

There were some questions where it was never or … not relevant and I’m thinking … what did not relevant mean? … you might think not relevant is fairly self-explanatory but it’s not in my case … what’s not relevant? … I’ve ticked it and it’s not relevant because it never occurred … [so] you’d say never, wouldn’t you? PID9, Patient with psoriasis, UK

The edits to items 12 (“I have struggled to concentrate”) and 26 (“I have been prevented from or found it difficult to be intimate with another person”) reduced the need for the not relevant option as these items could now apply to all respondents, regardless of employment or relationship status. This option was no longer necessary and was therefore removed to simplify.

## 4. Discussion

This study tested the content validity, acceptability, and feasibility of PRIDD. It met the highest standards for cognitive interviews outlined by COSMIN (Supplementary material 10) (19, 27), providing high-quality evidence of the comprehensibility, comprehensiveness, and relevance of PRIDD from the target population. The study findings and resultant adjustments produced a 26-item version of PRIDD, ready for field testing, and psychometric testing.

### 4.1. Implications for measuring the impact of dermatological conditions on the patient’s life

#### 4.1.1. The challenge of dermatology-specific PROMs

With the International Classification of Disease (ICD)-10 (52) classifying over 1,000 dermatological conditions, dermatology patients are a particularly heterogeneous group in relation to age and condition type, relative to other medical specialties. Unsurprisingly then, participants differed in their relation to the term “dermatological condition” but understood the rationale behind this and no alternate sufficiently inclusive terms were suggested.

While PRIDD appears to be relevant to people with dermatological conditions overall, some sub-group differences were found. The physical, psychological, and social impacts were generally consistent across conditions but practical impacts such as time and financial resources differed for people with alopecia. They emphasized the positive impacts of their condition, for example, regaining time lost to styling hair. Nevertheless, the feedback indicated a consensus that PRIDD was relevant and accepted across conditions. We believe this shows that, despite their inherent challenges, dermatology-specific PROMs are appropriate but need to be developed carefully with high levels of patient involvement throughout.

#### 4.1.2. The conceptual framework of impact

The findings of this cognitive interview study support our conceptual framework of the impact of dermatological conditions (26). First, participants’ lived experiences encompassed the biopsychosocial nature of their conditions. Second, no new items or domains were added to PRIDD, and participants could identify which items corresponded to the underlying domains. Third, the data support our previous decision to remove an “impact of healthcare” domain from the original conceptual framework (37). One participant, for example, summarized this decision while reflecting on being dismissed by healthcare professionals saying, “that could be a whole … paper all by itself … that’s a whole different ball game if you get involved in that” PID14. Future work should quantitatively hypothesis test the conceptual framework to complete the evaluation of content validity.

Given the importance patients placed on the influence of healthcare in determining the impact of their condition in our previous work (26, 37), we suggest that these data could form the basis of a separate ‘quality of dermatological care’ measure.

#### 4.1.3. Patient perspectives on issues with response options

We pilot tested a 5-point rating scale with an additional NRR option for each item. NRR options are common in dermatology. The DLQI, the most widely used PROM in dermatology (53), for example, uses a sum score of its 10 items, eight of which have a NRR option that is given the same zero score as “not at all” responses (22). This scoring method assumes that NRRs are due to a lack of disease burden and therefore have no impact on overall quality of life scores. However, recently several independent studies have shown that this interpretation is problematic (54–56) and revised scoring methods have subsequently been proposed (54, 57). Concurrent with these findings, we found the NRR to be problematic as participants differed in their interpretation of this option with consequences for the accuracy of their scores.

This calls into question the current use and scoring of NRRs in dermatology PROMs. Indeed, the emerging body of research on NRRs has shown that approximately 20%–76% of patients provide at least one NRR on the DQLI (54, 56, 58–60). The frequency of NRRs differs across socio-demographic groups with the elderly, females, those not working full time, and less educated patients reporting higher rates of NRRs than others (54). This is may be related to the consistent finding that the DQLI items with the highest rates of NRRs are those on impairment in work and school, sport, sexual relationships, and social activities (54–56, 61, 62), areas of life that may be less applicable to these particular subgroups. Content validity requires that the PROM is comprehensive, comprehensible, and relevant *across* the target population. Such high frequency and differing rates of NRRs suggest content validity issues with the DLQI as certain items are not relevant to a significant proportion of patients and subgroups groups (31). Indeed, since relevance is a central parameter of content validity (19, 27), the inclusion of items with NRRs, regardless of their rates, could be viewed as a fundamental threat to content validity.

Because NRRs are scored as 0, a higher yield of NRRs should be associated with a lower DLQI score. However, Rencz et al. (54) found the inverse; a greater number of NRRs was associated with a higher DQLI score, indicating poor construct validity. Researchers and clinicians have hypothesized that NRRs do not reflect actual lower quality of life burden but rather the opposite.

To our knowledge, this is the first study to provide the patient perspective on NRRs and clarify the above findings. First, we provide qualitative data to show that socio-demographic factors do play a role in the use of NRRs. For example, the item on work and study was not relevant to people who were retired. Second, our data suggest that low disease burden is not the only reason patients chose an NRR option. As hypothesized previously, NRRs can indicate a high disease burden. This is most clearly seen in items relating to romantic relationships and intimacy. Some participants felt that these items were not relevant to them as they were not in a relationship but explained that their condition was the cause of this. These findings challenge the use of NRRs in dermatology PROMs as they show that the assumption of low disease burden is not always correct.

We overcame the issues inherent in NRRs by following our participants’ recommendation to remove this option. As all the items pilot tested here had been prioritized for inclusion in PRIDD, it was clear that these were important impact concepts to patients and therefore we did not remove items to remove the NRR. Instead, we maximized the applicability of each item across the target population. For example, the item “It has been hard to work or study” was changed to “I have struggled to concentrate” as this tapped the underlying concept while being applicable regardless of employment status or age. We were also careful not to link items too closely to specific examples. To illustrate, the DLQI item on leisure and daily activities asks “how much has your skin interfered with you going shopping or looking after your home or garden?.” The true impact of the condition on the patient’s leisure and daily activities may be hidden by this question if shopping, housework, or gardening are not relevant to them. We used neutral wording, e.g., “my leisure time/activities have been negatively affected” to overcome this. We also reworded the item “It has been difficult to be intimate with a partner” to “I have been prevented from or found it difficult to be intimate with another person” to increase its relevance in light of the finding that NRRs to this item may be due to high disease burden. We believe that by removing the NRR option and rewording items to maximize their applicability across the target population, we have overcome the scoring limitations of the existing dermatology PROMs by using a more valid and reliable method.

#### 4.1.4. Determining the recall period

Choice of recall period is an aspect of internal validity as a suboptimal recall period can introduce measurement error. There is no “gold standard” recall period for PROMs as “one size does not fit all” (63). The FDA guidance (64) on PROM development states a preference for items with short recall periods or those that ask patients to describe their current or recent state. Their rationale is twofold. First, longer recall periods are thought to undermine content validity because they rely on memory and therefore may introduce recall bias. Second, longer recall periods may be impractical in research or clinical practice with frequent data collection points or clinic visits due to overlapping periods. Hence, we initially assumed that a one-week recall period would be the most appropriate for PRIDD.

Study participants almost unanimously criticized the one- and two-week recall periods and proposed longer recall periods (e.g., 1–6 months, years, or lifetime), supporting the concept of Cumulative Life Course Impairment in dermatology (65–68). Participant’s views strengthen previous work suggesting that a shorter recall period likely underestimates the burden of long-term conditions (69), particularly those with a relapsing-and-remitting course (70), results in loss of information (71) and that patients can accurately recall over a longer period of time than the FDA guidance suggests (36), particularly when their issues are bothersome and memorable (69, 72). In response, we changed the recall period to 1 month, which is within the recommended range for PROMs of phenomena such as quality of life and is likely to reduce the risk of recall bias (63).

#### 4.1.5. Acceptability and feasibility of PRIDD

During PROM development, a balance is evident between maximizing the information gained about the construct of interest and reducing respondent burden, meaning that every item in a PROM must earn its place (21). Where participant interviews demonstrated that items were not easily interpreted or clearly aligned with their underlying concept of interest, they were removed or edited during the interview rounds. In this way, this study provides evidence of the value of each of the 26 items.

PRIDD appears to be feasible for use, with participants taking an average of approximately 4 min to complete. The average time to complete PRIDD in research and clinical practice is likely to be lower because, in most cases, participants were thinking and responding aloud while completing PRIDD and edits were consequently made to improve the comprehensibility of the measure.

### 4.2. Strengths and limitations

Though it is an important step in the development and validation of new PROMs (27), the pilot testing of dermatology-specific PROMs is rare and, when conducted, is often of poor methodological quality (17). This is the first pilot study of a dermatology-specific PROM to both (a) be of high methodological quality and (b) show evidence sufficient content validity, acceptability, and feasibility, according to the COSMIN criteria (27).

The main strength of this pilot test is the use of qualitative methods. The interviews followed the COSMIN-recommended TSTI method of cognitive interviewing (38), eliciting data from a range of sources (i.e., observational, think aloud, and probing techniques). Our approach of asking participants to elaborate in detail regarding their understanding of each aspect of and item in PRIDD provided manifold definitions and examples of impact as well as identifying how participants understood each item. From these data, we could detect and resolve problems with item wording, ordering, and redundancies. Notably, we followed the COSMIN guidance by testing each aspect of PRIDD separately and in its final form, except for a minor adjustment to the instructions (27). PRIDD, therefore, is the first dermatology-specific PROM to meet the COSMIN standards for cognitive interviews (17).

Participants were sampled purposively through GlobalSkin’s unique global network to achieve diversity in terms of clinical (e.g., common and uncommon, inflammatory and non-inflammatory dermatological conditions) and demographic variables (e.g., age and gender). This strengthens the content validity of PRIDD for global use across dermatological conditions. However, our participants were mainly patient organization members and therefore may not represent the experiences or views of non-members. With a sample size of 12, we were able to demonstrate data saturation as no major problems that could be resolved were identified in the final round of interviews. Still, rarer problems or those pertaining to conditions not represented may have gone undetected. Five of the 12 participants had psoriasis and we were unable to recruit participants with other common conditions such as acne, eczema, and vitiligo or those who do not speak British, American, or Canadian English, reducing the transferability of PRIDD. The next stage of development is psychometric testing. Here, we will be able to further test PRIDD with a larger sample of patients representing a wider array of dermatological conditions and global regions and who speak other variations of English.

### 4.3. Implications for clinical practice

A PROM’s potential to advance person-centered care is contingent upon its applicability, comprehensiveness, and relevance to patients. PRIDD should not *replace* the discussion of the wider impact of the disease during the clinical consultation, but *facilitate* and frame patient-centered discussions (73). Indeed, participants in the current study and previous dermatology PROM development work [e.g., (74)] expressed a desire to provide qualitative information alongside their response options to afford clinicians a deeper understanding of their lived experiences.

As Tourangeau’s (40, 41) cognitive theory demonstrates, the completion of a PROM requires cognitive processing. Clinicians should be aware that a patient’s literacy level, among other factors, may facilitate or create barriers to PROM completion. Consequently, patient-centeredness in the administration of PROMs should be paramount to avoid perpetuating health inequalities.

Consistent with the literature (75, 76), while developing PRIDD, participants have consistently expressed that the psychological aspects of dermatological conditions require more attention (26, 37). There is a need to establish effective psychological interventions and pathways to psychological support, improve clinicians’ skills and confidence to address psychological and social issues (77) and develop effective collaboration between dermatologists and mental health professionals including psychologists.

### 4.4. Implications for research

Some participants reported positive impacts of their dermatological conditions and expressed a desire for these to be measured as well as the negative impacts. The positive impacts reported—e.g. empowerment, confidence, and gratitude—are congruent with Tedeschi and Calhoun’s (78) work on post-traumatic growth and validate the analysis of our concept elicitation study which also discerned positive impacts (26). Because PRIDD focuses on the burden of dermatological conditions, positive impacts were not incorporated as this would violate the unidimensionality required of the measure. Given the importance of the various positive impacts to patients, the data gathered throughout the development of PRIDD could serve as the basis of a new, separate measure of the positive impact of dermatological conditions. Qualitative research to inform the development of psychological interventions typically focuses on the negative aspects of long-term health conditions, but it can be worthwhile to consult with people with positive experiences as they are well placed to provide input that may lead to effective interventions.

Researchers, clinicians, and regulatory agencies should choose measurement instruments based on their quality. Before PRIDD can be recommended for use in research and clinical practice, validation of the measurement properties (validity, reliability, and responsiveness) and interpretability information (i.e., Minimally Important Change) is required (36). While cognitive interviews allow patients to have greater input into the item refinement process than purely statistical methods allow, ideally, they would not be the sole method of item refinement. Several items were identified as having conceptual overlap in the current study. It will be important to test for item redundancy and data structure in the psychometric testing phase.

## 5. Conclusion

In this final step in the content validity phase of development, PRIDD was pilot tested through cognitive interviews with the target population. The data triangulated previous findings, recommendations, and the conceptual framework of impact. The results provide insight into how patients understood the items in PRIDD and shed light on the patient perspective on current debates regarding PROMs in dermatology. The resultant confirmation of the comprehensibility, comprehensiveness, relevance, acceptability, and feasibility of PRIDD provides evidence of content validity from the target population. The next step in the development of PRIDD is the psychometric testing phase.

## Data availability statement

The datasets presented in this article are not readily available because, due to the nature of this research, participants of this study did not agree for their data to be shared publicly. Requests to access the datasets should be directed to RP, pattinsonr@cardiff.ac.uk.

## Ethics statement

This study involves human participants was reviewed and approved by Cardiff University School of Healthcare Sciences Ethics Committee. The patients/participants provided their written informed consent to participate in this study.

## Author contributions

RP: conceptualization, methodology, validation, formal analysis, investigation, resources, data curation, writing—original draft, writing—review and editing, visualization, supervision, and project administration. NT-S: methodology, validation, and writing—review and editing. RH, MV, and NT: investigation and writing—review and editing. JA: conceptualization, resources, writing—review and editing, project administration, and funding acquisition. AF: resources, writing—review and editing, and project administration. NC: methodology, writing—review and editing, and supervision. MA: conceptualization, methodology, writing—review and editing, and supervision. CB: conceptualization, methodology, validation, resources, writing—review and editing, and supervision. All authors contributed to the article and approved the submitted version.

## Funding

This study was funded by GlobalSkin as a part of its Global Research on the Impact of Dermatological Diseases (GRIDD) project.

## Conflict of interest

The authors declare that the research was conducted in the absence of any commercial or financial relationships that could be construed as a potential conflict of interest.

## Publisher’s note

All claims expressed in this article are solely those of the authors and do not necessarily represent those of their affiliated organizations, or those of the publisher, the editors and the reviewers. Any product that may be evaluated in this article, or claim that may be made by its manufacturer, is not guaranteed or endorsed by the publisher.
