# Supplementary material for: Evidence of the content validity, acceptability, and feasibility of a new Patient-Reported Impact of Dermatological Diseases measure
Source: Front Med (Lausanne). 2023 May 5;10:1020523. doi: 10.3389/fmed.2023.1020523 (PMC10196461; doi:10.3389/fmed.2023.1020523)
Supplement: Supplementary file 1 [file Data_Sheet_1.docx]

Supplementary Material

# Supplementary Material 1: Schematic diagram of the development and validation of PRIDD


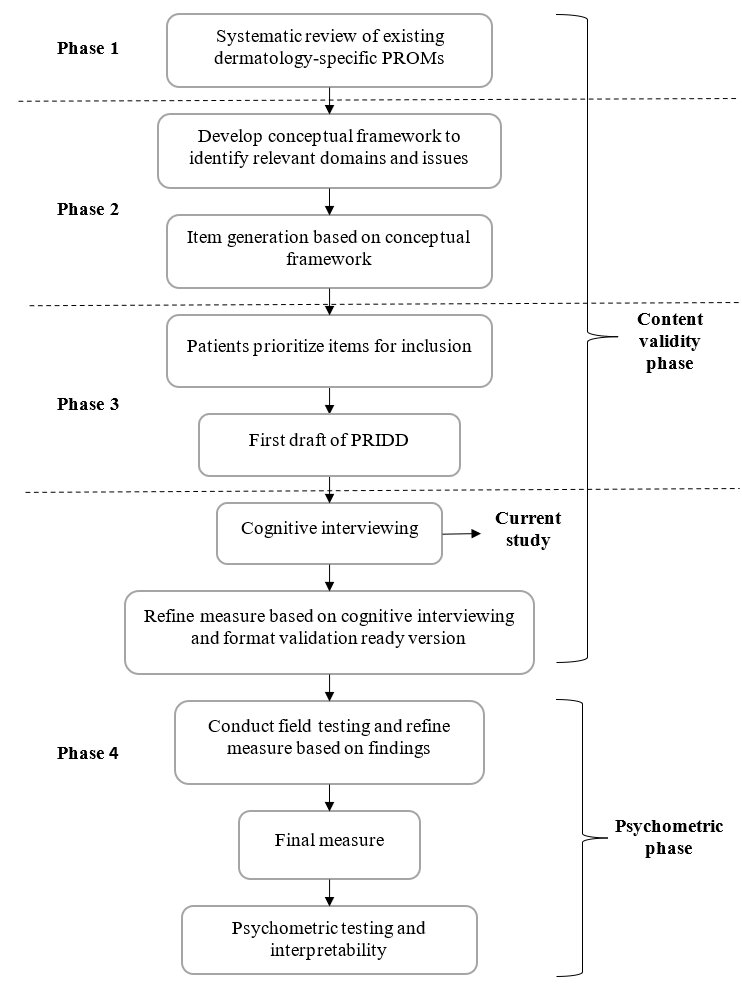


# Supplementary Material 2: Excerpt of PRIDD (first draft)

**Patient-Reported Impact of Dermatological Disease**

We know dermatological conditions impact people over a long period of time, but the aim of this questionnaire is to measure how much your dermatological condition has affected your life OVER THE LAST WEEK. Please mark one box for each question. If a statement does not apply to you, for example because you do not work, please mark “not relevant”. NOTE: The term ‘skin’ here includes the mucous membrane.

**Because of my dermatological condition…**

|  |  | Never | Rarely | Sometimes | Often | Always | Not relevant |
| --- | --- | --- | --- | --- | --- | --- | --- |
| 1 | ...the quality, look or feel of my skin/hair/nails has bothered me |  |  |  |  |  |  |
| 2 | …I have experienced physical discomfort, soreness or irritation |  |  |  |  |  |  |
| 3 | …my skin has been sensitive |  |  |  |  |  |  |
| …  DRAFT | | | | | | | |
| 9 | …my everyday choices have been affected (for example, choice of clothes, hair style or products) |  |  |  |  |  |  |
| 10 | …I have struggled to perform roles important to me (for example, to be caregiver / parent/ partner) |  |  |  |  |  |  |
| 11 | …my leisure time/activities have been negatively affected |  |  |  |  |  |  |
| 12 | …it has been hard to work or study |  |  |  |  |  |  |
| 18 | …I have felt angry, annoyed or frustrated |  |  |  |  |  |  |
| … | | | | | | | |
| 19 | …I have felt like I’ve lost some control |  |  |  |  |  |  |
| 20 | …I have felt embarrassed |  |  |  |  |  |  |
| 21 | …I have felt unattractive |  |  |  |  |  |  |
| 22 | …I have felt dismissed or abandoned by others  DRAFT |  |  |  |  |  |  |
| … | | | | | | | |
| 26 | …it has been difficult to be intimate with a partner |  |  |  |  |  |  |
| 27 | …I have been excluded, bullied or discriminated against |  |  |  |  |  |  |

**Please make sure you have answered every question clearly. Thank you for helping us understand and measure the impacts of your dermatological condition.**

# Supplementary Material 3: Conceptual framework of the impact of dermatological conditions on the patients’ life


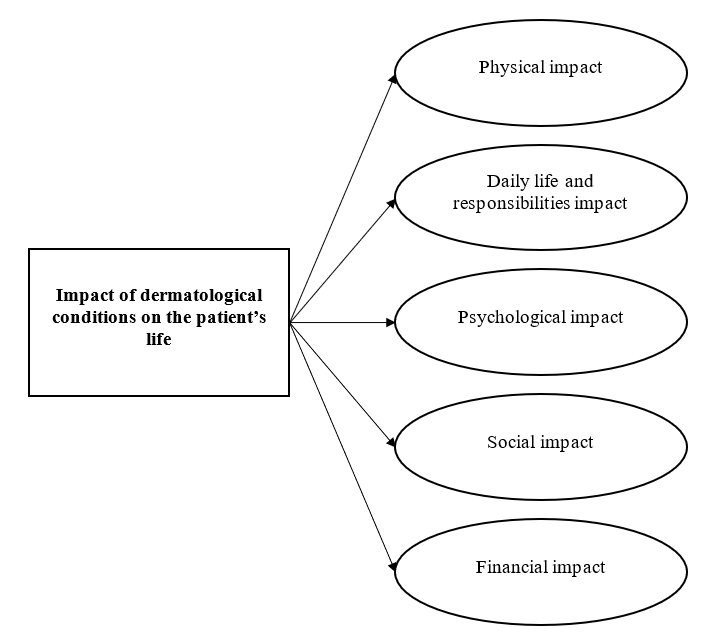


# Supplementary Material 4: Cognitive interview topic guide and its development

The cognitive interview questions were developed following guidance from the International Society for Pharmacoeconomics and Outcomes Research (ISPOR)(64) and Brod, Tesler and Christensen (46). Questions met the COSMIN standards for cognitive interviewing by asking participants about both the comprehensibility *and* comprehensiveness of PRIDD as well as assessing the instructions, items and response options separately (26). The topic guide was semi-structured to standardize the interviews while allowing the interviewer to probe the reasons or explanations for the participants’ responses. It was continually adapted to reflect new topics or themes that needed further probing. An item definition list accompanied the topic guide. The list stated the intended meaning of each item so that the interviewer could assess whether the participant understood the items as intended.

## Stage 1: Set-up (5 - 10 mins)

#### *Briefly introduce self*

#### *Overview of purpose and process of the cognitive interview*

Ask participant whether they have done a cognitive interview before. If not, explain cognitive interview process.

Before we begin, are there any questions you’d like to ask me?

I am sending you the link to PRIDD in the chat section:

- [Link to PRIDD (original)]
- [Link to PRIDD (reversed)]

Once the screen has loaded please could you share your screen with me. But please don’t start completing the questionnaire until I turn the recorder on.

Let participant know that we are beginning the interview and I will start recording.

#### Stage 2: Observation (5 - 10 mins)

Please start completing the questionnaire and let me know when you’ve finished.

Use timer to time how long it takes the participant to complete PRIDD.

Observe the respondent completing the questionnaire. Note facial expressions or indications of reading difficult. Listen for comments about difficulty reading or questions that indicate lack of clarity or ease of use.

#### Stage 3: Interview (30 - 50 mins)

1. **General questions**

- What did you think of the measure overall?
- Were any of the questions in any way offensive or objectionable to you?
- What did you think about the amount of time it took you to complete the questionnaire?
- What suggestions do you have for changing the questionnaire, so it is easier to complete?

1. **Instructions questions (refers to intro paragraph starting ‘We know dermatological conditions impact people over a long period of time’)**

- Can you tell me in your own words, what this instruction is asking you to do?
- Can you describe any confusion or difficulty you had in understanding these instructions?
- Are there any words or phrases that you would change to improve the instructions?

1. **Recall period questions**

- What period of time did you think about when you were completing the questionnaire?
- Does the time frame you were asked to think about when answering the questions allow you to easily answer the questions?
- When you completed the questionnaire, do you think you were able to accurately remember your experiences over the last two weeks?

1. **Items questions**

Ask the participant to go through the questionnaire and explain what each item means in their own words. Ask them to talk about what they thought about when they were answering the item and give a relevant example.

The participant may have already answered the item specific questions while completing the above exercise. If not, ask the following item specific questions.

***Item specific questions***

- Which of these questions do you think refers to the symptoms of your condition? (The answer we want is Item 1)
- Do you experience itch? If so, which of these questions do you think refers to itch? (The answer we want is Item 2)
- Item 4 When you say your sleep is being disturbed, are you finding it hard to go to sleep in the first place or do you find you wake up periodically during the night?
- Item 14 - (…my life goals and choices have been affected) – How well does this question work with the two-week recall period?
- Item 25 - (…it has been difficult to be intimate with a partner) - Is there a more acceptable way that we could word this item?

#### *Composite items questions (items 1, 2, 5, 15, 17, 18)*

- We’ve grouped them together, because we think they belong together. Otherwise, we’d have three separate questions. Which of those applies to you most?
- Do they fit together?

#### General prompts [No need to ask all of these]

- Using your own words, how would you explain what this question means?
- Is the question worded in a way that made sense to you?
- Was the question about something which is important or relevant to you?
- If you can, give me an example of when that question has applied to you.
- What do you think that question means? Can you put it in your own words?
- What was the key word in that question that meant something to you? Is that the correct word or do you think another word is more appropriate. What substitute word might you use for [key word]?
- This question seemed to be less straightforward than some of the others. Can you take me through that?
- You seemed to understand this question very easily. What was it about this question that you found easy to answer?

1. **Response options questions**

- I’m keen that we use the best scoring system. There isn’t one single way of scoring a response, but this is how we’ve decided to do it. I want you to tell me whether you found that easy or difficult and whether you have any advice for us about the scoring system.
- Do you understand the time differences between each option?
- What caused you to choose this response?

#### Stage 4: Ending (5 – 10mins)

We have now come to the end of our discussion.

Before we finish,

- Is there anything I forgot to ask?
- Is there anything else you would like to comment on regarding the survey?

Thank you all so much for taking the time to participate in this interview. Your opinions are very important in guiding the development of PRIDD and further research. You can track the progress of this research on GlobalSkin’s website where the research team will be posting updates.

# Supplementary Material 5: Elaboration on the three steps of the TSTI method followed in the cognitive interviews

Following general introductions, the three steps of the TSTI method were followed:

**Step 1:** The interviewer encouraged the respondent to ‘think out loud’ while completing PRIDD and observed (both visually and aurally) the respondent while reading, interpreting, and answering PRIDD. During this step, the interviewer did not comment but observed and took notes for use in steps 2 and 3. The interviewer also recorded the time taken to complete PRIDD.

**Step 2:** The interviewer clarified and supplemented the data gathered in step 1 by questioning the participant about their response behavior. The questions asked in the second step depended on the observations of step 1; for example, if the participant was observed to hesitate, the interviewer might ask “I saw that you hesitated when answering question 1, can you remember why you hesitated?’’

**Step 3:** The third and final step was an interview with in-depth probing to elicit experiences, explanations, interpretations, and opinions from the participant.

After the first six participants were interviewed (Round 1), the findings were reviewed by RP, who decided whether any changes to any aspects of PRIDD were required. Two further rounds of interviews we conducted to reach consensus from participants that PRIDD was appropriate and most problems were detected.

# Supplementary Material 6: Elaboration on the data analysis following the thematic analytical model of cognitive interviewing

Figure 1 illustrates each step and product in the analytical process. The downward pyramid on the left represents the reduction of data from the raw data of individual interviews to the thematic schema generated by the comparisons of interviews. The upwards pyramid on the right represents the progression and growth in understanding of item performance.

Figure 1: The steps in the thematic analytical model of cognitive interviewing and their products in terms of data reduction and knowledge generation


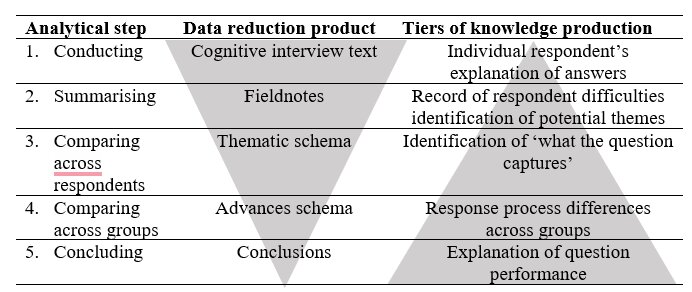


Steps one and two apply to the conduct of the interviews. In step three, all participant responses regarding a particular item were reviewed to identify common themes across participants using constant comparison techniques. In step four, subgroups were compared to identify whether any particular theme was more apparent among any specific group of respondents. This served to identify how participants’ experiences and social contexts affected item interpretation and responses. In the fifth and final step, the data were synthesized and how the item and subscales performed across participants were summarized.

# Supplementary Material 7: Summary of changes made to PRIDD between cognitive interview rounds

| **Part of questionnaire** | **Round 1** | **Round 2** | **Round 3** |
| --- | --- | --- | --- |
| **Instructions** | We know dermatological conditions impact people over a long period of time, but the aim of this questionnaire is to measure how much your dermatological condition has affected your life OVER THE LAST TWO WEEKS. Please mark one box for each question. **NOTE**: The term ‘skin’ here includes the skin, hair, nails and mucous membrane. | We know dermatological conditions impact people over a long period of time, but this questionnaire aims to measure how much your dermatological condition has affected your life **OVER THE LAST MONTH**. Please mark one box for each question. **NOTE**: The term ‘skin’ here includes the skin, hair, nails and the skin-like inner lining of organs and cavities (i.e. the mucous membrane). | We know dermatological conditions impact people over a long period of time, but this questionnaire aims to measure how much your dermatological condition has affected your life **OVER THE LAST MONTH**. Please **consider each question in relation to your dermatological condition**. **Mark one box** for each question. Dermatological conditions can affect the skin, hair, nails and/or mucous membrane. The word ‘skin’ here includes any of these aspects relevant to your condition. |
| **Recall period** | 2 weeks | 1 month | 1 month |
| **Number of response options** | 6 | 6 | 5 |
| **Response option wording** | Always / Often / Sometimes / Rarely / Never // Not relevant | Always / Often / Sometimes / Rarely / Never // Not relevant | Always / Often / Sometimes / Rarely / Never |
| **No. items refined** | 8 | 11 | 5 |
| **Item wording refinements** | ...the quality, look or feel of my skin has bothered me | …my general physical health has been negatively affected | … my skin has been sensitive to external factors (for example, to touch, light or temperature) |
|  | …my leisure time has been negatively affected | …my treatment has caused practical problems (for example, by taking up time or being messy) | …my life goals and choices have been affected (for example, career choice or having children) |
|  | …I have struggled to concentrate | …I have struggled to perform roles important to me (for example, to be caregiver / parent / partner / employee / student) | …my condition has dominated my thoughts |
|  | …I have had extra financial costs | …my leisure time (for example, hobbies, sports or exercise) has been negatively affected | …I have felt like I’ve lost some control over my life |
|  | …I have been distracted by my skin | …I have had extra medical or non-medical financial costs | …I have been prevented from or found it difficult to be intimate with another person |
|  | …I have felt low in mood or motivation | …my life goals and choices have been affected (for example, career choice) |  |
|  | ...my relationships with those close to me have been negatively affected | …I have struggled to think about anything other than my condition |  |
|  | …I have felt dismissed by others (including healthcare professionals) | …I have felt depressed or low in mood |  |
|  |  | …I have felt shame or embarrassment |  |
|  |  | …it has (or would have) been difficult to be intimate with another person |  |
|  |  | …I have been excluded, stigmatised or discriminated against by others |  |
| **No. items removed** | 0 | 1 | 0 |
| **Items removed** |  | …I have felt dismissed by others (including healthcare professionals) |  |

# Supplementary Material 8: Excerpt of the pilot tested version of PRIDD

**Patient-Reported Impact of Dermatological Diseases**

We know dermatological conditions impact people over a long period of time, but this questionnaire aims to measure how much your dermatological condition has affected your life **OVER THE LAST MONTH**. Please **consider each question in relation to your dermatological condition**. **Mark one box** for each question. We have provided examples to give you an idea of the things you might consider, but these should not limit your answers. Dermatological conditions can affect the skin, hair, nails and/or mucous membrane. The word ‘skin’ here includes any of these aspects relevant to your condition.

**Because of my dermatological condition…**

|  | DRAFT | Always | Often | Sometimes | Rarely | Never |
| --- | --- | --- | --- | --- | --- | --- |
| 1 | …the quality, look or feel of my skin has bothered me |  |  |  |  |  |
| 2 | …I have experienced physical discomfort, soreness or irritation |  |  |  |  |  |
| 3 | …my skin has been sensitive to external factors (for example, to touch, light or temperature) |  |  |  |  |  |
| 4 | …my sleep has been disturbed |  |  |  |  |  |
| …. | | | | | | |
| 9 | …my leisure time (for example, hobbies, sports or exercise) has been negatively affected |  |  |  |  |  |
| 10 | …my everyday choices have been affected (for example, choice of clothes, hairstyle or products) |  |  |  |  |  |
| 11 | …my life goals and choices have been affected (for example, career choice or having children) |  |  |  |  |  |
| 12 | …I have struggled to perform roles important to me (for example, to be caregiver / parent / partner / employee / student) |  |  |  |  |  |
| ….  DRAFT | | | | | | |
| 18 | …I have felt shame or embarrassment |  |  |  |  |  |
| 19 | …I have felt unattractive |  |  |  |  |  |
| 20 | …I have been hiding, covering or concealing my condition |  |  |  |  |  |
| 21 | …I have struggled to concentrate |  |  |  |  |  |
| …. | | | | | | |
| 25 | …I have been prevented from or found it difficult to be intimate with another person |  |  |  |  |  |
| 26 | …I have been excluded, stigmatised or discriminated against by others |  |  |  |  |  |

**Please make sure you have answered every question. Thank you for helping us understand and measure the impacts of your dermatological condition.**

# Supplementary Material 9: Summary of the evidence of comprehensibility, relevance and detected problems for each item

| Original item | Evidence of comprehensibility and/or relevance | Reported problems | Final item after revision |
| --- | --- | --- | --- |
| The quality, look or feel of my skin/hair/nails has bothered me | I'd be looking at it in two … in two respects … my head, which I'm bald, … and then I'd also be thinking about my wigs and how they look (15) Patient with alopecia, UK | Inclusion of ‘skin/hair/nails’ added complexity to the item. | The quality, look or feel of my skin has bothered me |
| I have experienced physical discomfort, soreness or irritation | It’s a question that does apply to me and my condition. It is discomfort, soreness and irritation … all three would apply to me. (12) Patient with extensive linear porokeratosis, Ireland  I would immediately read it as itching, putting it in a different way. (13) Patient with psoriasis, UK | N/A | I have experienced physical discomfort, soreness or irritation |
| My skin has been sensitive | Sensitive I would understand as it would react easily to certain products if … you’re limited to skincare and moisturisers, lotions, that’s everything, shampoo, conditioner, body wash, so a reaction to products, easily inflamed, and an easy reaction … That it would really invoke a reaction of some sort if it comes into contact with something. (12) Patient with extensive linear porokeratosis, Ireland | Participants descriptions demonstrated conceptual overlap with the previous item:  Has it been itchy? (13) Patient with psoriasis, UK | My skin has been sensitive to external factors (for example, to touch, light or temperature) |
| My sleep has been disturbed | If your sleep's affected, you don't rest properly, and you're tired and it’s like everything's a chore. You know, and that's a good measure, if somebody's not well, they're not sleeping, they're up, they're itching … good question, I like it. (1) Patient with psoriasis, UK | N/A | My sleep has been disturbed |
| I have felt tired, fatigued or lacked energy | You're going to feel tired, fatigue and lack of energy, and that’s as a result of your condition. (12) Patient with extensive linear porokeratosis, Ireland | N/A | I have felt tired, fatigued or lacked energy |
| My general health has been negatively affected | I interpret that as my overall health and how my condition has had an impact on my overall health and if I’ve suffered any other … ill health due to the condition that I currently have … it does have a knock-on effect on my general health … your condition sometimes will lead you to have to take certain medication, like really strong medication, which has side effects … if you do have a condition your general health is affected because you’re at higher risk, so with my condition, I’m at higher risk of developing skin cancer. … it depends what type of feedback you’re trying to get, and it could be that it’s physical general health versus mental general health, but other way, I combine everything together. (12) Patient with extensive linear porokeratosis, Ireland | Participants were unclear whether this item pertained to physical or mental health or both.  I just sort of read that as it affected my health in any way really, sort of general, physical, mental health … general health, I don’t think is a great term … is a bit vague. (15) Patient with alopecia, UK | My general physical health has been negatively affected |
| My preferred daily routine has been negatively affected | I read that as, you know, if my condition had made any impact negative, on my daily routine … as it says on the tin. (15) Patient with alopecia, UK | N/A | My preferred daily routine has been negatively affected |
| My treatment has caused problems (for example, by taking up time or being messy) | I interpret the question as has caused problems, as anything that you, as a patient, would see as a problem. So I wouldn’t limit it to just taking up time or being messy – the examples that you provide … I would say it has caused problems … way more than just taking up time or being messy … like being expensive, being frustrating. (12) Patient with extensive linear porokeratosis, Ireland | Participants with alopecia felt this item was less relevant to them.  No treatments, not applicable (18) Patient with alopecia, UK | My treatment has caused practical problems (for example, by taking up time or being messy) |
| My everyday choices have been affected (for example, choice of clothes, hair style or products) | If you had not given the example or choice, clothes, hairstyle, products, if you had just given about my everyday choice, the first thing my brain would connect is choice of clothes, and choice of hairstyle and products … for me, they’re prioritised in that way. (12) Patient with extensive linear porokeratosis, Ireland | N/A | My everyday choices have been affected (for example, choice of clothes, hairstyle or products) |
| I have struggled to perform roles important to me (for example, to be caregiver / parent / partner) | If all of a sudden, my skin and my joints start to really seize up, I couldn't have walked my mum to the car this morning, I couldn't. I'd struggle, between us, my mum can't open a bottle of milk, and if … my hands have gone bad, I can't open a bottle of milk … I think that's actually an important question … even an example of maybe like a parent and a partner … if somebody's married, and … they're going down … it'll affect their partner … Because they're going to have to step [up] and do more, they'll have to do the treatment side of it. (1) Patient with psoriasis, UK | With the changes to the item ‘I have struggled to work or study’, this item was changed to include struggling to perform as an employee or student. | I have struggled to perform roles important to me (for example, to be caregiver / parent / partner / employee / student) |
| My leisure time/activities have been negatively affected | Avoiding swimming, certain sports … I quite enjoy swimming … Other people’s reaction in those days … some people … take offence by the look of you … and that affects you, you know, when you’re fairly … self-conscious. Other people’s reaction has an effect, so, you know, I avoided swimming. (3) Patient with psoriasis, UK | Participants were unclear as to whether leisure time included social time.  I think they could be one question, yeah. It's social life and leisure time, they're pretty much the same thing, I think. My leisure time is my social life basically, so I think they could be one question. (1) Patient with psoriasis, UK | My leisure time (for example, hobbies, sports or exercise) has been negatively affected |
| it has been hard to work or study | Has the skin irritation made me lack concentration on what I’m doing or trying to do … I’ve retired and consequently that’s why I have to sort of cast my mind back a little bit to answer that question. The sort of things I might be concentrating these days aren’t really as important as they would have been in the past, so you know if I’m trying to concentrate on doing the Sudoku, I wouldn’t really think twice about my psoriasis but perhaps trying to concentrate on an exam question you suddenly become very aware of how it’s irritating you. (13) Patient with psoriasis, UK | Participants understood this as assessing their ability to concentrate. The word ‘struggle’ was preferred over ‘hard’.  I don't know whether that'd be better phrased in a similar way to number 10, sort of struggle to perform work and study, duties or things, but obviously it's the same sort of answer. But I mean, it was straightforward and yeah, you're sort of asking whether or not it impacted your work or your study. (15) Patient with alopecia, UK | I have struggled to concentrate |
| I have had extra out-of-pocket expenses | I have had extra medical and non-medical financial costs, yes, exceedingly so … £300 I waste every month [on wigs] … that's 300 times like 12 times 23 years, it's a lot of money when you add it up, so it's like mortgage money we're talking. (18) Patient with alopecia, UK | Participants were unsure of how to interpret the phrase ‘out-of-pocket expenses’:  If I’d asked the question, I would have said I have had extra costs rather than out of pocket expenses … out of pocket expenses to me sort of suggests … immediate cash expenditure rather than you know costs which come from anywhere. (13) Patient with psoriasis, UK  Some participants did not consider medical expenses when responding to this item:  I suppose I find extra out of pocket expenses outside of my regular doctor bills or regular medical bills … they would be my standard bills. And then everything extra out of pocket would be, you know, when something goes wrong and you need bandages, plasters, scissors, medical kits, creams, ointments. (12) Patient with extensive linear porokeratosis, Ireland | I have had extra medical or non-medical financial costs |
| My life goals and choices have been affected | I wanted to be a teacher, and just never got there, through one thing and another, and hospitals and illness … shame that question, but it's good it's there. (1) Patient with psoriasis, UK  That’s pretty self-explanatory. You know, because you do: you have to think of your disease, you know, in the moment and in the long-term. (14) Patient with psoriasis, Canada | Some participants were unsure whether professional choices should be considered when responding to this item. | My life goals and choices have been affected (for example, career choice or having children) |
| I have felt anxious, worried or nervous | “I have felt anxious, worried or nervous.” Again, that’s very easy to understand and very real. That’s a real question. I like that one. (14) Patient with psoriasis, Canada | N/A | I have felt anxious, worried or nervous |
| I have been preoccupied with my skin, hair or nails | It's something that I think about regularly, but is it better to think about it regularly, or am I distracted by it, am I not able to think about other things … I'm thinking about that instead of other things and can't sort of stop thinking about it. (15) Patient with alopecia, UK | Participants felt the word preoccupied was not right:  [Keep] dominated in there, it's better that preoccupied, preoccupied doesn't even touch the sides’ (18) Patient with alopecia, UK | My condition has dominated my thoughts |
| I have felt down, blue or low in motivation | It’s a very relevant question. My answer is: often – and I think it’s a good choice of words. For me, a synonym for all of them perhaps would be ‘depression’. (5) Patient with hidradenitis suppurativa, Ireland | ‘Low in motivation’ was generally associated with items 5 (I have felt tired, fatigued or lacked energy) or 24 (my social life or interactions have been negatively affected), rather than low mood. It was suggested that ‘low motivation’ was removed from the item:  I suppose feeling down and blue is different to low in motivation, to me sometimes, as well. Because you know, you can be low in motivation for a lot of other reasons than feeling low in general… you could maybe just remove the low in motivation, if that's captured in energy levels, like fatigue. (15) Patient with alopecia, UK  Participants also suggested including the word ‘depression’ in the item, as it was not clear whether the item was intended to capture this extreme end of the spectrum of low mood, in the way that item 15 does with anxiety.  If the question is determined to … quantify the level of depression, I would probably add the word depression in there. Same as anxiety, the word anxiety is in a different question, so that, you know, people that suffer from anxiety, they have panic attacks, they may need like anti-anxiety medication, same as depression … by not putting it in there … it’s going to be kind of seen as that it’s been overlooked or that you maybe don’t want to say the word depression … Question 17) probably needs to be rephrased to link it more to depression, because it’s, it’s probably, er, a little bit deeper than just being down. (12) Patient with extensive linear porokeratosis, Ireland | I have felt depressed or low in mood |
| I have felt angry, annoyed or frustrated | I knew what was being asked … if I felt anger, frustration or annoyance, I don't really think there's any other way to ask that. (15) Patient with alopecia, UK | N/A | I have felt angry, annoyed or frustrated |
| I have felt like I’ve lost some control | Well, it controls every aspect of your life … it dictates what you do, it dictates what you wear, it dictates where you go … it dictates everything … when it's bad. (16) Patient with PRP, UK | It was not clear what control referred to:  You’ve lost some control which would cover a lot of aspects of work. Your personal, your social … angry, frustrated … you covered a lot of that … I suppose it all depends how you read into the question. (11) Patient with discoid lupus, Ireland | I have felt like I’ve lost some control over my life |
| I have felt embarrassed | There has been embarrassment with regard to the smell … I have felt embarrassed with clothing – staining has come through on clothing and people have said, ‘Have you sat on something, or did you spill something.’ (5) Patient with hidradenitis suppurativa, Ireland | Participants felt the word shame was important to include in this item:  The only thing I would add there is, shame, I think shame is … an important feeling (12) Patient with extensive linear porokeratosis, Ireland | I have felt shame or embarrassment |
| I have felt unattractive | I have felt unattractive. Often, is my answer to that. It’s a very, very relevant question. (5) Patient with hidradenitis suppurativa, Ireland | N/A | I have felt unattractive |
| I have felt dismissed or abandoned by others | I’ve frequently been told, ‘You should be familiar, or used to it by now.’ I feel very dismissed that things that are important to me are just not taken seriously … you hope in a friendship, or in any type of relationship, that people will give proper consideration to things that are important to you, and that doesn’t happen. Yeah, you feel somewhat abandoned in a relationship, whether it’s an intimate relationship, or friendship, or professional relationship, etc. (5) Patient with hidradenitis suppurativa, Ireland | Participants found that this item was not easy to understand. Most participants could not distinguish between this item and item 27 (I have been excluded, bullied or discriminated) as they considered it to be alluding to social exclusion or workplace discrimination.  I read as being kind of excluded from a group or from you know something. (13) Patient with psoriasis, UK | Item removed |
| I have been focused on hiding, covering or concealing my condition | Great question … people do cover and hide it. Which makes it worse … I like that question … I wouldn’t change anything to it. I understand it fully. (14) Patient with psoriasis, Canada | N/A | I have been focused on hiding, covering or concealing my condition |
| My social life or interactions have been negatively affected | Social life would be interacting with friends, doing things outside of the home, participating in community work or interacting with others. Interactions could be just going about your day-to-day business, interacting with society: going shopping, going to the dentist – your everyday things. Yes, they have been negatively affected. As mentioned, I try and minimise my interaction with others. (5) Patient with hidradenitis suppurativa, Ireland | N/A | My social life or interactions have been negatively affected |
| My relationships with others have been negatively affected | I’d say often my relationships, intimate relationships, family relationships, friends, professional relationships and so on, it’s a very relevant question … I like the fact that it’s just left at relationships and the patient can interpret that in whatever way they want … Friends will be reluctant to ask me to do things because of this. I’m reluctant to participate and engage fully in relationships, because of my dermatological condition. (5) Patient with hidradenitis suppurativa, Ireland | Participants acknowledged some conceptual overlap with the previous item but generally felt that these items with important and distinct enough to retain both:  I think it's important, I think [keep] both because I think affecting your relationships, it's more specific, and I think … it's shown a greater impact than if it's just affecting you socially… if it's actually affecting personal relationships that you have, then I think they are separate things. (15) Patient with alopecia, UK | My relationships with those close to me have been negatively affected |
| It has been difficult to be intimate with a partner | I think that's also a really relevant question … it's something I've experienced in the past … it should be there. I know it's probably a more personal question, and people might struggle to answer it, but I think it's important because it goes into your relationships, and if that affects you, that's a big impact on your life … there's only so many ways you can reword something … I don't know how you can ask it in a more delicate way than what you already are, so yeah. (15) Patient with alopecia, UK | Participants echoed criticisms of relationship and intimacy items in other dermatology-specific PROMs because, in Round 1, this item was only relevant to people who had a relationship or active sex life:  This is the question that I always struggle with, not relevant because I've not got a partner… the reason why I've not got a partner is because it's been difficult … I think it's an important question … [it’s] probably the reason why I'm single, in my formative years between when I started getting psoriasis and in hospital, was years when all my mates were getting wives and babies and all that. All of a sudden it had just passed me by, it had gone (1) Patient with psoriasis, UK | I have been prevented from or found it difficult to be intimate with another person |
| I have been excluded, bullied or discriminated against | I have been excluded, bullied, or discriminated against. My answer to that would be: sometimes. Excluded is perhaps most relevant for me here … I’ve been excluded from things by friends, by family, etc. Because I am restricted in what I can do physically, etc. Bullied … it doesn’t apply to me … I think excluded is very, very relevant, very poignant and ... Bullied or discriminated, perhaps when I was younger, when I was a teenage boy, participating in teen sports. Yeah, there would have been comments passed, because of physical marks on my skin, etc., etc. (5) Patient with hidradenitis suppurativa, Ireland | The appropriateness of ‘bullied’ was questioned as participants felt that it was relevant to childhood rather than adult experiences. | I have been excluded, stigmatised or discriminated against by others |

# Supplementary Material 10: Evaluation of the quality of the pilot test against the COSMIN standards for cognitive interviewing

| **COSMIN standard** | | **Requirement** | **Rating** | **Details** |
| --- | --- | --- | --- | --- |
| 14 | Was a cognitive interview study or other pilot test conducted? | A cognitive interview study or other pilot test performed to test the PROM for comprehensibility and comprehensiveness | **Very good** | A cognitive interview study was conducted. |
| 15 | Was the cognitive interview study or other pilot test performed in a sample representing the target population? | Study performed in a sample representing the target population | **Very good** | Participants were drawn from PRIDD’s target population of adults worldwide living with a dermatological condition. Purposive sampling was employed to achieve a sample with maximum variation according to participants’ dermatological condition and demographic factors (e.g. age, gender, and country of residence). Table 1 demonstrates that the sample represented the target population. |
| ***Comprehensibility*** | | | | |
| 16 | Were patients asked about the comprehensibility of the PROM? | Patients asked about the PROM instructions, items, response options, and recall period | **Very good** | Participants were asked about the comprehensibility of PRIDD’s instructions, items, response options, and recall period. |
| 17 | Were all items tested in their final form? | All items were tested in their final form | **Very good** | All items were tested in their final form. Minor edits were made to the instructions in the third round of interviews (see Supplementary Material 7). |
| 18 | Was an appropriate qualitative method used to assess the comprehensibility of the PROM instructions, items, response options, and recall period? | Widely recognized or well justified qualitative method used | **Very good** | The Three-Step Test-Interview method was employed to test the comprehensibility of PRIDD’s instructions, items, response options, and recall period separately. |
| 19 | Was each item tested in an appropriate number of patients? | ≥7 patients | **Very good** | Each item was tested by 12 participants. |
| 20 | Were skilled interviewers used? | Skilled group moderators/interviewers used | **Very good** | All interviews were conducted by a skilled interviewer (RP, RH or ML). All interviewers had experience in conducting dermatological and qualitative research and were trained in cognitive interviewing techniques. |
| 21 | Were the interviews based on an appropriate interview guide? | Appropriate topic or interview guide | **Very good** | A topic guide, including the semi-structured interview guide, was developed to structure interviews (see Supplementary Material 4). The interview questions were developed based on ISPOR and other guidance. The guide was continually adapted to reflect refinements made to PRIDD or new topics or themes that needed further probing. |
| 22 | Were the interviews recorded and transcribed verbatim? | All group meetings or interviews were recorded and transcribed verbatim | **Very good** | Each interview was recorded using a Dictaphone. Audio data were subsequently transcribed verbatim by a Cardiff University approved independent transcription provider. During the observation step of the interviews, interviewers made notes on facial expressions or comments. |
| 23 | Was an appropriate approach used to analyze the data? | A widely recognized or well justified approach was used | **Very good** | Analysis followed the thematic analytical model of cognitive interviewing. |
| 24 | Were at least two researchers involved in the analysis? | At least two researchers involved in the analysis | **Very good** | Two researchers (RP and NTS) were involved in the data analysis. RP independently coded the data. The analysis was checked by NTS, and issues were discussed and resolved. |
| 25 | Were problems regarding the comprehensibility of the PROM instructions, items, response options, and recall period appropriately addressed by adapting the PROM? | No problems found or problems appropriately addressed, and PROM was adapted and re‐tested if necessary | **Very good** | After each round of interviews, PRIDD was adapted to address problems found and re-tested in the next round of interviews. |
| ***Comprehensiveness*** | | | | |
| 26 | Were patients asked about the comprehensiveness of the PROM? | Patients asked whether the items together comprehensively cover the construct the PROM (or subscale) intends to measure. | **Very good** | Patients were asked whether the items together comprehensively cover the impact of dermatological conditions. |
| 27 | Was the final set of items tested? | The final set of items was tested | **Very good** | All items were tested in their final form. No edits were made to PRIDD during the fourth and final round of interviews (see Supplementary Material 7). |
| 28 | Was an appropriate method used for assessing the comprehensiveness of the PROM? | Widely recognized or well justified method used | **Very good** | The Three-Step Test-Interview method was employed to test PRIDD’s comprehensiveness. |
| 29 | Was each item tested in an appropriate number of patients? | ≥7 patients | **Very good** | Each item was tested by 12 participants. |
| 30 | Were skilled interviewers used? | Skilled interviewers used | **Very good** | All interviews were conducted by a skilled interviewer (RP, RH or ML). All interviewers had experience in conducting dermatological and qualitative research and were trained in cognitive interviewing techniques. |
| 31 | Were the interviews based on an appropriate interview guide? | Appropriate topic or interview guide | **Very good** | A topic guide, including the semi-structured interview guide, was developed to structure interviews. The interview questions were developed based on ISPOR and other guidance. The guide was continually adapted to reflect refinements made to PRIDD or new topics or themes that needed further probing. |
| 32 | Were the interviews recorded and transcribed verbatim? | All group meetings or interviews were recorded and transcribed verbatim | **Very good** | Each interview was recorded using a Dictaphone. Audio data were subsequently transcribed verbatim by a Cardiff University approved independent transcription provider. During the observation step of the interviews, interviewers made notes on facial expressions or comments. |
| 33 | Was an appropriate approach used to analyse the data? | A widely recognised or well justified approach was used | **Very good** | Analysis followed the thematic analytical model of cognitive interviewing. |
| 34 | Were at least two researchers involved in the analysis? | At least two researchers involved in the analysis | **Very good** | Two researchers (RP and NTS) were involved in the data analysis. RP independently coded the data. The analysis was checked by NTS, and issues were discussed and resolved. |
| 35 | Were problems regarding the comprehensiveness of the PROM appropriately addressed by adapting the PROM? | No problems found or problems appropriately addressed, and PROM was adapted and re‐tested if necessary | **Very good** | After each round of interviews, PRIDD was adapted to address problems found and re-tested in the next round of interviews. |
| Overall score | | | **Very good** | |
